# Tennessee Dental Association

**Published:** 1868-10

**Authors:** Wm. M. R. Johns

**Affiliations:** Rec. Sec'y.


### CORRESPONDENCE.

---

### ARTICLE III.

### *Tennessee Dental Association.*

The Second Annual Meeting of the Tennessee Dental Association was held at Dr. Arrington's Office in the City of Memphis on the 21st, 22nd, and 23d, days of July 1868. A large number of invitations having been freely circulated among the Dentists of Tennessee and adjoining States, there was a goodly number present representing the profession from such sections of the country as have not heretofore for many years realized the beneficial and happy influences consequent upon associated effort.

The association was called to order with President Wm. H. Morgan in the Chair and Wm. T. Arrington Secretary.

The roll was called and minutes of last meeting read and approved.

The various official reports were received and filed.

Reports of committees received and committees discharged.

The Secretary read a number of communications from absentees and others.

Committee on Membership presented the following applications, all of whom were duly elected members by ballot.

Dr. Wm. M. Johns, of Sommerville; Dr. L. Angspath, of Hellina; Dr. E. W. Sawyer, of Memphis; Dr. V. F. Elliott of Memphis; S. Hinson, of Memphis; Dr. Wm. Wasson, of Memphis; Dr. T. F. Linde, of Memphis; Dr. E. S. Chisholm, of Tuscumbia; J. L. Mewborn, of Bolivar.

On motion the regular order of business was suspended, and the following essays were read—

| | |
|---|---|
| Dental Physiology, | by W. D. Tucker. |
| Microscopical Anatomy, | " Wm. T. Arrington. |
| Pathology, Histology & Dent. Med. | " Wm. R. Johnston. |
| Metallic Plates, | " G. W. Acree. |
| Vulcanite Rubber Plates, | " E. C. Ford. |
| Dental Chemistry, | " M. McCarty. |
| Appliances for regulating teeth, | " J. B. Wasson. |
| Dental Education, | " J. A. Arrington. |
| Diseases of the Teeth, | " H. M. Acree. |

The following Officers were elected to serve tor the ensuing year.

| | | |
|---|---|---|
| President, - | Wm. T. Arrington, - | Memphis. |
| 1st Vice " - - | S, J. Cobb, - - - | Nashville. |
| 2nd " " - | G. W. Acree, - - - | Memphis. |
| Rec. Secretary, - - | Wm. M. R. Johns, - | Sommerville. |
| Cor. " - | Wm. H. Morgan, - - | Nashville. |
| Treasurer, - | E. C. Ford, - - - | Sommerville. |
| Executive Committee | M. McCarty, | Pulaski. |
| | S. Hinson, | Memphis. |
| | Wm. Wasson, | Memphis. |

The newly elected officers having been duly installed, the retiring President, Dr. Morgan, delivered an able address upon the origin, progress and present status of the Dental Profession, a copy of which was ordered to be sent to the American Journal of Dental Science for publication.

On motion it was unanimously resolved that the next Semi-annual meeting be held in the City of Nashville, commencing on the 16th of December 1868 and the Annual meeting to be held at Lookout Mountain on the Tuesday preceding the last Tuesday in July 1869, to both of which the members of the profession throughout this and adjoining States are respectfully invited to attend and take part in the various discussions.

The second and third days of the meeting were consumed in discussing the following subjects, To wit;

1st. Diseased Gums and Alveolar Abscess.
2nd. Treatment of Exposed Pulps.
3rd. Best means of Obtinding Sensitive Dentine.
4th. Best diet and most effective method of promoting the developement of osseous structure.
5th. Filling Approximate Cavities.
6th. Dental Education.
7th. Miscellaneous Subjects.

Unfinished business was taken up, and the President appointed the following Essayists for the next meeting:

| | | |
|---|---|---|
| Dr. G. W. Acree, | on | Taking Impressions. |
| " L. Angspath, | " | "Cleansing & Polishing Teeth." |
| " S. Hinson, | " | "Sulp. Ether as an Anæsthetic." |
| " M. McCarty, | " | " Facial Neuralgia." |
| " V. F. Elliott, | " | " Treatment of Exposed Pulps." |
| " Wm. H. Morgan, | " | Conservative Dentistry. |
| " J. B. Wasson, | " | Diagnosing diseases of the Oral Cavity. |
| " S. P. Cutler, | " | Physiology. |
| " H. M. Acree, | " | Alveolar Abscess. |

Drs. J. B. Wasson, Wm. H. Morgan and V. F. Elliott were appointed a committee to draft resolutions relative to the death of Dr. J. P. Wilson, a member of this association.

Various committees were appointed, and the Association adjourned to meet at Nashville the 16th of December next.

Wm. M. R. Johns, *Rec. Sec'y.*

---

